# Train the brain with music (TBM): brain plasticity and cognitive benefits induced by musical training in elderly people in Germany and Switzerland, a study protocol for an RCT comparing musical instrumental practice to sensitization to music

**DOI:** 10.1186/s12877-020-01761-y

**Published:** 2020-10-21

**Authors:** Clara E. James, Eckart Altenmüller, Matthias Kliegel, Tillmann H.C. Krüger, Dimitri Van De Ville, Florian Worschech, Laura Abdili, Daniel S. Scholz, Kristin Jünemann, Alexandra Hering, Frédéric Grouiller, Christopher Sinke, Damien Marie

**Affiliations:** 1 Geneva School of Health Sciences, Geneva Musical Minds Lab (GEMMI Lab), University of Applied Sciences and Arts Western Switzerland HES-SO, Avenue de Champel 47, 1206 Geneva, Switzerland; 2 Faculty of Psychology and Educational Sciences, University of Geneva, Boulevard du Pont-d’Arve 40, 1205 Geneva, Switzerland; 3 Institute for Music Physiology and Musicians’ Medecine, Hannover University of Music, Drama and Media, Neues Haus 1, 30175 Hannover, Germany; 4 Center for Systems Neuroscience, Bünteweg 2, 30559 Hannover, Germany; 5 Center for the Interdisciplinary Study of Gerontology and Vulnerability, University of Geneva, Switzerland Boulevard du Pont d’Arve 28, 1205 Genève, Switzerland; 6 Department of Psychiatry, Social Psychiatry and Psychotherapy, Section of Clinical Psychology & Sexual Medicine, Hannover Medical School, Centre of Mental Health, Carl-Neuberg-Str. 1, 30625 Hannover, Germany; 7 Swiss Federal Institute of Technology Lausanne (EPFL), Route Cantonale, 1015 Lausanne, Switzerland; 8 Faculty of Medecine of the University of Geneva, Switzerland, Campus Biotech, Chemin des Mines 9, 1211 Geneva, Switzerland; 9 Swiss Center for Affective Sciences, University of Geneva, 1205 Geneva, Switzerland. Campus Biotech, Chemin des Mines 9, 1202 Geneva, Switzerland

**Keywords:** Music induced brain and behavioral plasticity, Age-related cognitive decline, One-year music practice, Randomized controlled trial, Working memory, Executive functions, Magnetic resonance imaging (MRI), Voxel based Morphometry (VBM), Diffusion tensor imaging (DTI), Multivariate data-driven analyses

## Abstract

**Background:**

Recent data suggest that musical practice prevents age-related cognitive decline. But experimental evidence remains sparse and no concise information on the neurophysiological bases exists, although cognitive decline represents a major impediment to healthy aging. A challenge in the field of aging is developing training regimens that stimulate neuroplasticity and delay or reverse symptoms of cognitive and cerebral decline. To be successful, these regimens should be easily integrated in daily life and intrinsically motivating. This study combines for the first-time protocolled music practice in elderly with cutting-edge neuroimaging and behavioral approaches, comparing two types of musical education.

**Methods:**

We conduct a two-site Hannover-Geneva randomized intervention study in altogether 155 retired healthy elderly (64–78) years, (63 in Geneva, 92 in Hannover), offering either piano instruction (experimental group) or musical listening awareness (control group). Over 12 months all participants receive weekly training for 1 hour, and exercise at home for ~ 30 min daily. Both groups study different music styles. Participants are tested at 4 time points (0, 6, and 12 months & post-training (18 months)) on cognitive and perceptual-motor aptitudes as well as via wide-ranging functional and structural neuroimaging and blood sampling.

**Discussion:**

We aim to demonstrate positive transfer effects for faculties traditionally described to decline with age, particularly in the piano group: executive functions, working memory, processing speed, abstract thinking and fine motor skills. Benefits in both groups may show for verbal memory, hearing in noise and subjective well-being. In association with these behavioral benefits we anticipate functional and structural brain plasticity in temporal (medial and lateral), prefrontal and parietal areas and the basal ganglia.

We intend exhibiting for the first time that musical activities can provoke important societal impacts by diminishing cognitive and perceptual-motor decline supported by functional and structural brain plasticity.

**Trial registration:**

The Ethikkomission of the Leibniz Universität Hannover approved the protocol on 14.08.17 (no. 3604–2017), the neuroimaging part and blood sampling was approved by the Hannover Medical School on 07.03.18. The full protocol was approved by the Commission cantonale d’éthique de la recherche de Genève (no. 2016–02224) on 27.02.18 and registered at clinicaltrials.gov on 17.09.18 (NCT03674931, no. 81185).

## Background

In young people, musical instrumental training triggers functional and structural brain plasticity and may enhance general cognitive and perceptual-motor function, explained by the widely distributed brain areas involved in music performance that support many other cognitive and perceptual-motor skills [1–12]. Evidence accumulates that this also applies to healthy elderly, at least in the behavioral domain [13–16].

We postulate showing advantages of music education for faculties generally acknowledged to decay during ordinary aging: executive functions, different types of memory, processing speed, language function, logical thinking, hearing in noise (auditory selective attention), manual dexterity, bimanual coordination, and to provoke functional and structural brain plasticity. Last but not least, we anticipate increased well-being. Optimal aging cannot be defined solely by objective factors such as mental performance and health, but also by subjective features such as quality of life [17]. Intriguingly, these objective and subjective factors appear to be closely related (for a review see [18]). The ultimate objective, if our study results confirm our hypotheses, is systematic implementation of government-funded musical practice facilities in elderly centers and nursing homes.

### Age-related cognitive decline in healthy elderly

Whereas cross-sectional studies indicate a definite decrease for different cognitive functions from early adulthood onwards, longitudinal studies however only demonstrate marked deterioration for most functions after midlife. In the majority of healthy older adults, executive functions decline [19, 20], and its sub-processes such as working memory updating, inhibition and task-switching (cognitive flexibility) all gradually deteriorate with age [19]. Actually, decrease of working memory function, a fundamental component of general cognition, may explain several of the other higher order cognitive aging phenomena, like for instance deterioration of executive functions [21, 22]. In a lifespan perspective however [23–25], development during aging results from the combination of decline of certain physiological and cognitive functions, with the lifelong prospect of evolving through learning and interventions.

### Brain aging in healthy elderly

Unraveling brain processes that underlie cognitive decline symptoms, and developing training regimens that stimulate neuroprotection mechanisms to delay or even reverse those symptoms, may represent a key to potential remedies [19, 20]. Cognitive regression in healthy elderly people follows local structural and functional brain modifications such as reduction of prefrontal cortex and hippocampal gray matter (GM) and loss of anterior white matter integrity and functional connectivity [26–29]. Global connectivity however, i.e. networks of long-range cortical fiber structural connections (white matter), and functional connections (as measured by resting state functional MRI (fMRI; functional Magnetic Resonance Imaging) carrying information flow dynamics throughout the whole brain [30–32], critical for cognitive functioning, also becomes less performant in the aging brain.

Recently evidence accumulated that hippocampal function is vital to all types of relational memory, independently of the time span between learning and recollection [33, 34]. Atrophy of the hippocampus, in general occurring in late adulthood [19], provokes deterioration of working memory and long term memory, decrease of abstract thinking (fluid intelligence) [35] and progressively increased risk for dementia [36].

Cognitive activities and physical exercise -in addition to treatment of general medical conditions- may delay age-related atrophy in the hippocampus, or even expand its size [36–38]. Musical practice can induce functional and structural plasticity in the anterior and middle part of the hippocampus, and these changes are accompanied by increased proficiency in musical tasks, working memory and fluid intelligence [4, 39–41].

### Countervailing age-related cognitive decline by means of interventions

Therapies based on real life experiences, like musical practice, dancing or playing chess, more naturally induce far-reaching transfer of learning as they are complex and variable compared to laboratory training [42, 43]. This illimited complexity allows progressive increase in difficulty, keeping learning challenging at all times. As these activities are pleasant for an average individual, they are intrinsically motivating and therefore easy to maintain over long periods of time, meanwhile increasing well-being.

Among a multitude of cognitive training research in the elderly, [21, 22, 44–46], only two used piano or keyboard training [13, 14], over periods of 6 and 4 months, in musically untrained healthy elderly between 60 and 85 years. In one study [13] the piano group significantly improved in executive function and working memory, compared to a passive control group. Another more recent study [14] also showed improved executive function and enhanced well-being, and also a trend for motor advantages, as compared to a control group performing other leisure activities. In yet another study in healthy elderly (mean age 77 years) 15 weeks of drumming and singing improved verbal and visual memory as compared to literature training [16].

### Musical training as a model for behavioral and cerebral plasticity

As musical performance relies on widely distributed brain areas involved in many other cognitive and perceptual-motor skills [6, 7, 47–51], musical instrumental training provides a strong means of improving cognitive and perceptual-motor function provoking functional and structural brain changes. Most studies on brain and behavioral plasticity induced by musical practice concerned cross-sectional studies in young adults with differing levels of musical practice from childhood. Progressive music intrinsic and overall cognitive advantages in combination with widespread functional and structural brain plasticity occurred as a function of training intensity in auditory, limbic, perceptual-motor and higher order cognitive networks [1, 3–6, 9, 11, 40, 52–58].

Brain areas most susceptible to age-related cognitive decay, in inferior frontal and temporal cortices and their interconnecting white matter tracts, are strongly involved in musical instrumental practice and share neural substrates with language functions [7, 9, 47, 53, 55, 59, 60], working memory [5, 7, 53, 55, 59, 60] and broad-spectrum cognitive function, including executive functions (see next sub-chapter) [3–5, 55, 61]. A study comparing elderly mono- and dizygotic twins, of whom only one practiced music, demonstrated that musical instrumental practice, controlling for sex, education-level and physical activity, reduced the probability of dementia and other age related cognitive impairments [62]. Overall, cognitive decline and dementia can be postponed by recent and past musical activities [63, 64]. Music-supported therapy also proved effective in restoration of motor skills in elderly suffering from decline of motor function following stroke [65, 66]. Increased gray matter in qualified musicians as compared to less proficient musicians or non-musicians could be consistently demonstrated in the inferior frontal cortex [1, 3, 55, 67], the hippocampus [4, 39, 40, 68], and the entorhinal cortex that projects to the hippocampus [69]. Common age-related shrinking of inferior frontal cortices appeared absent in older male orchestra musicians [67]. A recent review article acknowledges music training as a promising approach for age-related cognitive decline [70]. Strikingly, even people practicing music moderately and exclusively during their childhood, experienced advantages for neural timing in speech perception at an advanced age [71].

### Far transfer effects in young and older people following music training

Far transfer effects for higher-order cognitive functioning following musical training occurred in adult musicians for verbal (auditory & visual modality) and tonal working memory [4, 6, 58], verbal long-term memory [72], executive control [73, 74], fluid intelligence [4, 75], visuo-spatial ability [55, 63] and attention [61]. Specifically in healthy elderly far transfer after musical training showed for executive function, attention, working memory, verbal and visual memory, sensorimotor function and subjective well-being [13, 14, 16]. Musical practice sharpens auditory attention and hearing in noise in children, young and elderly adults [61, 76, 77], both recurrent problems in normal aging. Perceptual-motor skills, that decline with advanced age, may also profit [14] and interact with higher order cognitive functions [78]. The triple auditory, visually and sensory feedback on the precision of motor performance yielded during musical practice may explain this observation.

### Intervention studies provoking brain and behavioral changes in elderly and older adults

Not only were older adults (mean age 60 years) able to learn three-ball cascade juggling over 3 months, moreover their gray matter increased transiently in the left hippocampus, bilateral nucleus accumbens and associative visual areas, quite similarly as in young adults [79]. Four weeks of video action gaming in elderly (60 to 85 years) modified frontal task related electroencephalography) and changes persisted after a 6 months delay [80]. Aerobic training in elderly induced volume increase in the prefrontal cortex and anterior hippocampus after 6 to 12 months of training [36, 81], and white matter integrity improved in frontal, temporal and parietal tracts [29]. In mature adults (≈35–40 years of age), increase in volume of the posterior hippocampus occurred, after acquiring their taxi drivers license in London, thus learning by heart the map of the city [82]. Piano tuners, also trained in advanced adulthood, showed volume increase in the anterior hippocampus and frontal operculum [83]. Learning to read musical notation over 3 months induced specific effects on visuo-spatial skills and on the function of the fusiform gyrus and superior parietal areas in initially musically naïve adults [84, 85]. Five-week musical training of adult musical novices sufficed to induce functional audio-motor (temporal-frontal) coupling, thus brain plasticity [86–88]. Specifically in elderly, 4 to 6 months piano training enhanced executive functions and working memory, compared to control groups [13, 14]. In another study involving healthy elderly, drumming and singing could improve verbal and visual memory [16]. Moreover, learning a new skill engages neural plasticity more strongly in older adults [89]. Also specifically in elderly, advantages of intensive new skill learning for memory function manifested [90].

We conclude that piano training in elderly musical novices may induce functional and morphological brain plasticity in the auditory and prefrontal cortex, hippocampus and elsewhere in the brain, as well as in long- and short-range white matter tracts and functional connectivity, together with concomitant cognitive and sensorimotor advantages, even at an advanced age. Intensive music listening and learning may already provoke a subset of these cognitive advantages.

Although decline of certain physiological and cognitive functions during aging is inevitable, brain and behavior remain plastic from cradle to grave. Therefore, lifelong education for seniors should become a significant policy focus in the framework of population ageing [79].

## Methods / design

For the sake of clarity and brevity, a great deal of comprehensive information concerning Methods and Design, is provided in the Tables:

Table 1: Trial Registration Data Set (according to WHO guidelines), p. 6

Table 2: Prescreening procedure in order to verify inclusion/exclusion criteria, p. 8

Table 3: Documents: information & consent forms, agreements, p. 9

Table 4: Behavioral Test Battery (psychometric tests), p. 10

Table 5: (f)MRI measurements, p. 11

Table 6: Study procedure with timelines, p. 12

List of Abbreviations, p. 15

**Table 1:** Trial Registration Data Set (according to WHO guidelines)

Data category	Information^32^
Primary registry and trial identifying number	ClinicalTrials.gov Identifier: NCT03674931
Date of registration in primary registry	17.09.2018
Secondary identifying numbers	Study ID number: 81185
Source(s) of monetary or material support	Swiss National Science Foundation (SNSF no. 100019E-170,410) and Deutsche Forschungsgemeinschaft (DFG no.323965454)
Primary sponsor	SNSF and DFG: Lead Agency grant (two-countries study)
Contact for public and scientific queries	Prof. Clara E. James: clara.james@hesge.ch
Title	Train the Brain With Music: Brain Plasticity and Cognitive Benefits Induced by Musical Practice in Elderly People in Germany and Switzerland (TBM)
Countries of recruitment	Switzerland, Germany
Health condition(s) or problem(s) studied	Age-related cognitive decline
Intervention(s)	Experimental group: intensive piano learning over 1 year
	Active control group: musical culture learning over 1 year (history and listening)
Key inclusion and exclusion criteria	Ages eligible for study: 64 to 78 years Sexes eligible for study: both, Accepts healthy volunteers: only
	Inclusion criteria: Healthy right-handed volunteers, between 64 and 78 years of age, native French/German speakers. No regular musical practice over the lifespan. Only retired individuals may participate.
	Exclusion criteria: Impaired/not-corrected auditory or visual accuracy, neurological diseases in the present or the past, cardiovascular diseases, excessive hypertension, obesity, diabetes mellitus, beginning dementia, mild cognitive impairment, clinical depression
Study type	Interventional
	Allocation: randomized intervention model according to 4 stratification factors (age, gender, score at the COGTEL (global cognition test) and socioeconomic status). Groups are balanced for those factors. Single blind study (subject)
	Primary purpose: prevention of cognitive and sensorimotor decline in retired elderly
Date of first enrolment	May 2018
Target sample size	150
Recruitment status	Completed
Primary outcome(s)	Positive transfer effects from intensive piano training (intervention group) - as compared to listening and learning about music without practice (control group) - on age-related cognitive decline: for working memory, executive functions, speed of information processing, auditory selective attention (hearing in noise) and abstract thinking. Associated functional and structural brain plasticity may show in gray and white matter in temporal (medial and lateral), prefrontal and parietal areas. Less decline or increase of gray matter volume and activation change may show in the hippocampus, as well as improved functional networking in frontal areas during working memory tasks.
Key secondary outcomes	Benefits of fine perceptual-motor skills may also manifest in the intervention group as compared to the control group associated with functional and structural brain plasticity in sensorimotor areas. Subjective quality of life, verbal memory and hearing in noise may improve in both groups, the latter two associated with functional and structural brain plasticity in auditory and frontal areas. Epigenetic alterations may be induced by piano learning in relation with functional and structural imaging parameters.

### Research hypothesis

We intend exhibiting for the first time that musical activities, particularly musical instrumental practice, can countervail cognitive and perceptual-motor decline supported by functional and structural brain plasticity.

### Aims and objectives of the study

Our long-term aim is to promote healthy mental aging in elderly and therefore independence, autonomy and well-being through musical activities, specifically musical instrumental training of a complex instrument.

The preparatory phase of our study consisted in developing distinct musical interventions specifically designed for musically naive elderly aiming to support cognitive and sensorimotor development. Our study objective is investigating whether piano practice (intervention group) over one full year, as compared to listening and learning about music without practice (active control group), can provoke multiple far transfer effects on working memory, executive functions, speed of information processing, auditory selective attention (hearing in noise), abstract thinking, manual dexterity and bimanual coordination, all of which are strongly involved in piano practice and most relevant for independence and autonomy in everyday life. In association with these behavioral benefits we anticipate functional and structural brain plasticity in temporal (medial and lateral), prefrontal and parietal areas and the basal ganglia. We foresee potential benefits in both groups for verbal memory, hearing in noise and well-being, with more moderate cerebral plasticity in prefrontal, auditory and emotion regulation areas.

The ultimate goal, if our study results confirm our hypotheses, is systematic implementation of government-funded musical practice facilities in activity centers for elderly and nursing homes.

### Design

The study is a two-site Hannover-Geneva longitudinal randomized single blind intervention study in altogether ~ 155 retired healthy elderly (64–78) years, (63 in Geneva, 92 in Hannover, selected according to our inclusion/exclusion criteria out of ~ 500 applications), divided into 2 parallel groups, offering either piano training (intervention group) or musical sensitization training (active control group), both provided by professional musicians.

The active control group serves to control for music listening of similar styles, acquiring general knowledge, social interaction, attentional aspects, weekly instruction away from home, and daily homework.

All participants were informed before enrolling that the study aim is to compare two distinct music interventions and that both may have positive impact on cognitive functioning and brain plasticity (single blind procedure).

In order to prevent strong inhomogeneity between the groups, we use a blocked (“stratified”) randomized design, in order to prevent strong inhomogeneity between the groups at baseline. Stratification involved the factors age, gender, score at the COGTEL^1^ [95, 97] and socioeconomic status. After recruiting all participants, we applied a clustering procedure, separately at each site, to obtain the closest pairs of participants based on the stratification factors. We transformed the latter to z-scores and computed the Euclidian distance between participants, and finally, an algorithm attributed randomly 1 participant of each pair to each group. So, half of the participants ended up in the intervention group, and the other half in the control group at both sites.

Over 12 months participants receive weekly training for 1 hour (we aim at providing 40 courses over the 12 months, adapting to the holidays of the seniors), and also exercise at home for ~ 30 min per day. Participants committed not to miss more than maximum 8 courses on the full curriculum. Both groups study similar different music styles (classical music, world music, jazz, etc.). Subjects will be tested at 4 time points (0, 6, 12 months, and post-training at 18 months) on cognitive, perceptual and motor abilities (Behavioral Test Battery; Table 4), as well as via wide-ranging functional and structural neuroimaging assessments (Table 5).

Our research is inspired by Bugos and colleagues [13], but uses an extended methodology: 1) an active (vs passive) control group also learning music, 2) longer training (12 vs 6 months), 3) a narrower age bracket (64–78 vs 60–85 years of age), 4) extensive inclusion and exclusion criteria (for instance no prior musical education for more than 6 months (vs 5 years), 5) no major health problems, 6) a greater number of participants (150 vs 31), thus increased statistical power, and 7) more comprehensive psychometric testing involving cognitive, auditory, musical, sensorimotor and well-being measures. In addition, our study includes 8) comprehensive demographic, cognitive reserve index, sport leisure activities, well-being and food frequency questionnaires, as well as 9) multimodal (f)MRI brain imaging. Then 10) we also apply blood sampling, in order to perform genetic and epigenetic analyses with respect to music training and learning. Finally, 11) the piano groups will be trained in dyads, as group interaction is supposed to be specifically effective for learning in older adults [113].

### Setting

In Geneva (GE) all music courses and passage of the behavioral test battery take place at the Geneva School of Health Sciences (Haute Ecole de Santé de Genève (HEdS-GE) of the University of Applied Sciences and Arts Western Switzerland (HES-SO)). In Hannover (HA), Germany, music courses and passage of the behavioral test battery take place at the Hannover University of Music Drama and Media (HMTMH: Hochschule für Musik, Theater und Medien Hannover), and its Institute for Music Physiology and Musicians’ Medecine (IMMM: Institut für Musikphysiologie und Musikermedizin).

Professional musicians provide the music lessons, some of which are finishing their Master studies at the Geneva University of Music (Haute Ecole de Musique de Genève, HEM-GE) or at the HMTMH, but all already possess a Bachelor degree. Others are already established music/piano teachers and performers with a recently obtained Master’s degree. All music teachers possess several years of teaching experience prior to our study and were prepared to teach our specific population of elderly under the supervision of a team of music education specialists: Prof. Thomas Bolliger of the HEM-GE, and Prof. Wolfgang Zill and Prof. Andrea Welte in Hannover (HMTMH), who worked out a workflow together for both courses.

In Geneva, all brain imaging (MRI) is performed at the BBL, the Brain and Behavior Laboratory of the Geneva University. In Hannover all brain imaging takes place at the Hannover Medical school.

Registered nurses perform the blood sampling, in Geneva they are collaborators of the Geneva School of Health Sciences and in Hannover of the Hannover Medical School. Participants may refuse the blood sampling.

### Participants

The following criteria were verified by means of initial phone calls, a comprehensive demographic questionnaire, questionnaires on musical activities, tests on major hearing impairment, tests on cognitive functioning and clinical depression. A comprehensive list of all (pre) screening tests, that ensured the criteria were met, can be found in Table 2.

**Table 2:** Prescreening procedure in order to verify inclusion/exclusion criteria

PRESCREENING^a^	Action	Data collection
First contact	Telephone call; decision to proceed to Pre-screening Step 1	Age – gender – musical history – general health – native language – retirement – MRI compatibility – overall availability – contact information
PRE-SCREENING STEP 1		
Demographic questionnaire^b^	Send/receive by post	In-house questionnaire, detailed information on **languages**; **handedness; physical capability** to **come to the courses; health**: hospitalizations (causes), locomotion, vision (correction), audition (hearing aids), height & weight^c^, sleep quality; cardiovascular & neurological diseases, hypertension, diabetes, arthrosis, arthritis, rheumatism, bone fractures, thyroid problems, memory problems, other health problems; consent yes/no to contact the family doctor in case of fortuitous findings; **smoking** habits over the lifespan; **alcohol consumption** over the lifespan; present **medication**; other **drug use,** remarks; **sociodemographic information:** age, gender, nationality, civil status, siblings, children, highest level of education (5 categories), main professional engagements, gross monthly household income^d^ (5 categories); **MRI compatibility:** claustrophobia, implants; **Interest for the study:** motivation
Food Frequency Questionnaire FFQ^e^ [91]	Send/receive by post	Detailed information on food consumption (quality and quantity) over the last 4 weeks
Music questionnaires	Send/receive by post	Musical history (in-house questionnaire; GEMMI lab): amount and intensity of music listening and extracurricular music training: solfege/theory, instrumental training, choir singing, intracurricular music training in school Goldsmiths Musical Sophistication Index (GOLD MSI): self-report inventory and test battery for individual differences in musical sophistication (ability to engage with music) [92]
Godin-Shephard leisure-time physical activity questionnaire [93]	Send/receive by post	Adapted to cover different parts of life (2–25 y.o., last 5 years and last year)
In-house questionnaire on stressful life events	Send/receive by post	Self-report inventory on stressful events that may influence cognitive functioning, covering 6 time periods (12–25 y.o., 25–55 y.o, 55–64 y.o.; last 10 years, 5 years and year; GEMMI lab)
Geriatric Depression Scale^f^ [94]	Send/receive by post	Short version. Check for clinical depression
PRE-SCREENING STEP 2		
First laboratory visit - Basic clinical finger snap audition test - Psychometric tests i. Right-handedness ii. COGTEL^h^ [95]	Verify /complete questionnaires of Step 1 Pass 3 psychometric tests	Check for major hearing impairment^g^ Pass the Edinburgh Handedness Inventory (revised) to check for right handedness [96]; Measure gross overall cognitive level and check for potential (mild) cognitive decline (COGTEL)

^a^ Outliers (based on cut-off scores and or other exclusion criteria) for the tests of Pre-screening Step 1 & 2 are excluded from the study

^b^ All questionnaires are written in French and German; their translation was thoroughly verified by two bilingual researchers

^c^ Allows to compute the Body Mass Index (BMI), obese people are excluded (BMI > 30), as obesity impacts on brain (shrinkage) [133]

^d^ Adapted to Switzerland, France and Germany

^e^ Swiss Food Frequency Questionnaire, a semi-quantitative questionnaire existing in French and German, on 80 food items divided in 12 food groups

^f^ Geriatric Depression Scale short version

^g^ Refined information on hearing will be acquired during the Behavioral Battery, see Table 4 International Matrix Test

^h^ The COGTEL is a test of global cognition applied by telephone or face-to-face (we applied face-to-face). This hybrid cognitive test, developed for older adults by the team of Prof. Matthias Kliegel, provides a global measure of cognition, based on several memory and executive function subtests

Inclusion Criteria: good overall health; between 64 and 78 years of age; native or fluent French or German speakers; right-handed (for brain organizational reasons [114]); no regular musical practice over the lifespan (< 6 months); retirement.

Exclusion criteria: impaired/not-corrected auditory or visual accuracy; neurological diseases in the present or the past; mild cognitive impairment or beginning dementia (assured via the COGTEL score); cardiovascular diseases; excessive hypertension; obesity; diabetes mellitus; clinical depression; MRI incompatibility (f.i. implants or claustrophobia). Concomitant musical training during the study is prohibited (see Trust Agreement; Table 3).

In Geneva, Professor Giovanni Frisoni of the Medical Faculty of the University of Geneva, neurologist, specialist in the field of dementia, agreed to consult us and to receive participants showing signs of risk of mild cognitive impairment. In addition, Dr. Philippe Schaller, medical doctor, Head of Cité Générations, specialist in chronic diseases, agreed to consult us concerning general health issues of our elderly population.

In Hannover, Prof Eckart Altenmüller (neurologist) and Prof. Tillmann Krüger (medical doctor specialized in geriatric medicine and psychiatry), investigators on the study, will cover these issues.

Different documents: information & consent forms and agreements, were read and or signed by our participants, they are listed in Table 3.

**Table 3:** Documents: information & consent forms, agreements

TYPE OF DOCUMENT	When	What	Action	Who
Information on the study	During presentations; via flyers; “participant information form” (distributed or sent by post or mail)	Objectives of the study; Information on the inclusion/exclusion criteria; information on procedures and timelines; announcement of randomization within two different groups of music education; information on measurements and their frequency (*n* = 4): MRI, behavioral testing and blood sampling; information of reimbursing of travel expenses; telephone numbers and mails of the research team	Answer questions during presentations or by mail or telephone and during first lab visit	Research team
Declaration of informed consent	After inclusion in the study (following the 2 pre-screening steps see Table 2)	Consent to voluntary participate to the study with the right to withdraw at any time; accept the use of data for scientific and educational purposes, with assurance that the data will remain anonymous; to yes/no accept blood sampling (NOT an exclusion criterion); who to inform in case of potential unexpected detection of anomalies.	Read & Sign	Participant & member research team
Trust agreement	Before the courses start	The participant declares aiming to perform homework at least 30 min per day, 5 times per week, not to miss more than maximum 8 courses (on 40 in total), and announce absences and holidays well in advance with reasons and not to engage in any supplementary musical education	Read & Sign	Participant & member research team
MRI safety questionnaire	Before MRI measurements	Questions assuring MRI compatibility	Read & Sign	Participant & MRI operator
Debriefing on the fMRI task	After MRI measurements	Answer questions on sound perception, comprehension of the task, rating of difficulty of different experimental conditions, used strategies to perform the task, remarks	Read, answer questions	Participant & member research team
Homework questionnaire	3, 6, 9 & 12 months after the beginning of the music interventions	Time per day and per week spent on homework on average	Read and -sign	Participant

Consent to voluntary participate to the study involved: the right to withdraw at any time, accepting the use of the collected data for scientific and educational purposes –with the assurance that the data will remain anonymous-, to accept blood sampling or not -on a voluntary basis- and finally, whether to inform the participant or his or her treating physician in the event of unexpected potential detection of abnormalities.

## Interventions

### Music interventions

#### Intervention group: piano courses

Courses take place in dyads (2 participants together). Three Yamaha keybords P-45 B^2^ are set up in the classroom, with height adaptable piano stools. A Yamaha keybord P-45 B and a height adaptable piano stool are provided to each participant for use at home. During the courses the teacher sits in the middle, and in the beginning imitation and listening exercises are performed. Most of them are playful and allow the participants to familiarize themselves with the keyboard and adopt a correct and relaxed body posture. Clapping and singing or even walking within a certain rhythm are part of the lessons. Progressively music reading is introduced using a method specifically developed for our elderly population based on Jens Schlichting “Piano Prima Vista” (Internote GmbH Musikverlag 2013), and the Hall Leonard piano method for adults (ISBN: 9789043134378 & 9789043152037). Both methods exist in German and French. This material is enriched by different other pieces out of different textbooks (“A dozen a Day volume 1 (ISBN 9780711954311); Bastien Piano Basics - Piano Level 1 (ISBN-10: 0849752663; Manfred Schmitz Jugend-album für Klavier (ISBN: 978-3-932587-41-2), etc.), and sometimes by transcriptions of a favorite piece of one of the participants, arranged by the music teachers. Individual instruction is alternated with playing together, accompanied by the teachers. Sometimes one participant plays the right-hand part, while the other plays the left and vice versa. Homework includes practicing the exercises, the pieces and also occasionally composing music to perform during the course. A certain choice is left to the participants concerning the repertoire. Some improvisation is also introduced in association with basic notions of tonality and chords. The relationship between the teachers and the participants is informal.

#### Control group: musical culture^3^

Courses take place in groups of 4 to 6 individuals in order to create a stimulating environment. Lessons involve learning about the timbre and functioning of different musical instruments, music history, learning about composers, styles, etc. Presented music includes medieval, renaissance, classical, romantic and also modern classical repertoire, comprising orchestra and chamber music, opera, but also world music of all 4 continents and other styles (for instance music from the south Americas, jazz, pop, funk, etc.). During the highly interactive courses, the teacher explains the structure of the pieces, the role of the different instruments and voices and affective aspects of the pieces and develops a structured way of listening (“auditory analysis”) among the participants. During the lessons music related vocabulary may be written down, reassembling those terminologies in a notebook is part of the homework. Each week a voluntary participant presents music of his/her choice. The support material consists of extracts from textbooks, internet pages, documentaries, radio broadcasts, and presentations (f.i. “TED talks”, ted.com) and links for listening mainly of YouTube videos and audio material. Internet links and other materials for the upcoming course are provided in the week preceding the lessons. Homework involves resuming the course, listening to different musical pieces, reading texts and preparing presentations. Participants are continuously encouraged to express why he/she appreciates, or not, particular pieces or styles of music.

### Measurements and procedures

Subjects will be tested at 4 time points (t0: baseline, t1: after 6 months, t2: after training completion (12 months), t3: 6 months after training completion (18 months)) on 1) cognitive, perceptual and sensorimotor abilities by means of a battery of psychometric tests (Table 4) as well as via 2) wide-ranging functional and structural neuroimaging data (Magnetic Resonance Imaging MRI; see Table 5 and 3) Blood sampling.

**Table 4:** Behavioral Battery (psychometric tests) (measured at all 4 time points: t0: baseline; t1: 6 months; t2: 12 months & t3: 18 months)

BASIC COGNITION		
Processing speed & Attention	Digit-symbol	WAIS-IV, Wechsler, 2011 [98]
Short term memory	Forward Digit Span	WAIS IV, Wechsler, 2011
	Backward Digit Span	WAIS IV, Wechsler, 2011
**EXECUTIVE CONTROL**		
- Shifting	Number switch	Zuber et al., 2016 [99]
	Perceptual switch + Prospective Memory	Zuber et al., 2016
- Inhibition	GoDigit -NoGo	Enge et al., 2014 [100]
	Stop-Signal Task	Enge et al., 2014
**FLUID INTELLIGENCE**		
Logical reasoning	Matrices test	WAIS IV, Wechsler, 2011
**KNOWLEDGE**		
Episodic memory	Rey Auditory Verbal Learning Test	Bean, 2011 [101]; Rey, 1964 [102]; Helmstaedter et al., 2001 [103]
**MUSIC COGNITION**		
Musical aptitude	GOLD MSI (melody, rhythm) Harmony test (“Pietri”)	Müllensiefen, 2014 [92] Oechslin et al., 2013 [5]
**AUDITIVE COGNITION**		
Speech-in-noise perception	International Matrix Test (Oldenburg)	Kollmeier et al., 2015 [104]
**PERCEPTUAL MOTOR SKILL**		
Visuo-manual aptitude	Purdue Pegboard	Tiffin and Asher, 1948 [105]
Scale playing on keyboard	Scale analysis	Jabusch et al., 2004 [106]
**OTHER**		
WHOQOL-BREF^a^	Well-being	WHO, 2014^b^
Physical Activity Scale for Elderly	PASE (last two weeks activities)	Washburn, 1993 [107]

^a^World Health Organization Quality of Life-BREF (BREF for abbreviated)

^b^https://www.who.int/mental_health/media/en/76.pdf

#### Psychometric testing

A complete list of the psychometric tests is provided in Table 4.

#### Test-retest effects^4^

Test-retest effects are in principle corrected for by the existence of a control group.

However, most tests are modified from time point to time point, by

- Using different test material (stimuli) at each time point (COGTEL, Matrices test; Rey Auditory Verbal Learning Test, Harmony test (“Pietri”); GOLD MSI, International Matrix Test)
- Changing the order of the stimuli (Number Switch, Perceptual switch + Prospective Memory, Go-NoGo, Stop-Signal Task).

Only the tests: Digit-symbol and Digit Span forward and backward, and the sensorimotor tests Purdue Pegboard and Scale Analysis are repeated with an identical format at all time points. The questionnaire WHOQOL-BREF on subjective well-being is also repeated in identical format.

Finally, within time-points, the order of the tests is pseudo-randomized between individuals (as well as the lists of words in the International Matrix Tests).

#### (f)MRI measurements

Nota bene: the MRI measurements will not require any special preparation (such as having to drink or be injected with contrast materials or radioactive dye).

In Geneva the MRI measurements are performed on the imaging platform at the Brain and Behaviour Laboratory and benefit from support of the technical staff (BBL, http://bbl.unige.ch/), situated at the University Medical Centre of the University of Geneva, at close proximity to the HEdS-GE; with consent of the Director of the BBL, Patrik Vuilleumier, Full Professor at the Medical Faculty of the University of Geneva, neurologist and also Head of the Laboratory for Behavioral Neurology and Imaging of Cognition.

At both sites, 3 T Siemens scanners are used, however In Geneva acquisitions are performed on a 3 T whole body Siemens Trio system (Siemens TIM-TRIO, Erlangen, Germany) whereas In Hannover acquisitions are performed on a 3 T whole body Siemens Skyra system (Siemens MAGNETOM Skyra, Erlangen). At both sites, the scanners are equipped with an identical standard Siemens 32-channel head coil.

We adapted the sequences parameters, opting for a compromise between a good signal to noise ratio and a minimum amount of time. For the functional acquisitions, we adopted a multiband echo planar imaging fMRI with an accelerator factor of 3, allowing us to reduce the repetition time to 1.350 s which represents a good temporal resolution for fMRI (see Table 5). As a consequence of these choices, we could reduce the total duration of the MRI scanning to less than 1 hour, which is important given our elderly population.

The fact that 3 T Siemens scanners will be used at both sites already favors blending the data [115]. However, the challenge was to end up with matching MRI sequence parameters at both sites, allowing to pool the data with a minimum of post-hoc adaptations. We managed to end up with identical sequence parameters (see Table 5). Nevertheless, in all analyses, a “scanner” covariate will be included in order to disentangle scanner-effect from effects-of-interest.

A complete list of the different MRI measurements (*n* = 5) and main protocol parameters at both sites is provided in Table 5.

**Table 5:** MRI^a^ measurements (measured at all 4 time points: t0: baseline; t1: 6 months; t2: 12 months & t3: 18 months)

TYPE OF MRI MEASUREMENT / SCAN	Main technical details of the Siemens protocols
Grey matter volume assessment [108]	MP2RAGE^b^; duration: 8.22 min; voxel size: 1 mm isotropic; 176 slices; FOV^c^: 256x240x176 mm; TR^d^/TE^e^: 5000/2.98 ms; TI1^f^/TI2^g^: 700/2500 ms; flip angle 1/2: 4/5 degrees
Functional MRI Tonal working memory task [109]	EPI^h^; duration: 20.00 min; voxel size: 2.5 mm isotropic; 54 slices; FOV: 210x210x135 mm; multiband accelerator factor: 3; TR/TE: 1350/31.6 ms
Resting state functional MRI Allows to measure activity in the Default Mode Network (DMN) reflecting global functional connectivity of the brain [110]	EPI; duration: 10.31 min; voxel size: 2.5 mm isotropic; 54 slices; FOV: 210x210x135 mm; multiband accelerator factor: 3; TR/TE: 1350/31.6 ms
White matter assessment Allows to compute i.a. Fractional Anisotropy for evaluating white matter integrity reflecting structural connectivity [111].	DTI^i^; duration 7.38 min; voxel size: 1.5 mm isotropic; 84 slices; FOV: 222x222x126 mm; multiband accelerator factor: 3; TR/TE: 5163/109.2 ms; b-values: 0/1500 s/mm^2^; 65 directions, anterior to posterior phase encoding direction followed by a reverse direction for distortion correction; b-values: 0/ s/mm^2^)
Blood perfusion assessment [112]	ASL^j^; duration 6.10 min; voxel size: 2.5 × 2.5 × 3.0 mm; 36 slices; FOV: 220x220x129 mm; TR/TE: 3640/21.0 ms; flip angle: 90 degrees

^a^ MRI: Magnetic Resonance Imaging

^b^ MP2RAGE: Magnetization-Prepared 2 Rapid Acquisition Gradient Echoes

^c^ FOV: Field Of View

^d^ TR: Time to Repetition

^e^ TE: Time to Echo

^f^ TI1: Inversion Time 1

^g^ TI2; Inversion Time 2

^h^ EPI: Echo Planar Imaging

^i^ DTI: Diffusion Tensor Imaging

^j^ ASL: Arterial Spin Labeling

#### fMRI tonal working memory task

We use an in-scanner tonal working memory task (courtesy of Prof. Robert Zatorre and Dr. Philippe Albouy) [109]. We added a control condition to the original task that now comprises 3 experimental conditions, which can be used later on in different combinations for the fMRI contrasts in the analyses.

In all 3 conditions, 2 *pitch patterns* of 3 beeps (sinus tones, with a certain tone frequency or pitch) are played via MRI compatible earphones, with a *visual number code* displayed on the computer screen in between the 2 *pitch patterns*. In all conditions, the participant responds by pressing one of two buttons on a response pad (left vs. right).

In the ***simple condition***, 2 *pitch patterns* with 3 different pitches are played, thus forming kind of a melody, the first always followed by the *visual number code* 123 that represents the order of the notes of this first *pitch pattern* (1 for the 1rst note, 2 for the 2nd note and 3 for the 3rd note). The task of the participant is thus to respond whether the second pattern was identical to the first. If the order of the second *pitch pattern* is indeed identical, he/she should respond “correct” (left button). If the second *pitch pattern* is different (different order of the notes), they should respond “incorrect” (right button).

In the ***complex condition*** the trial sequence is the same, but the *visual number code* is never 123. It can be 321, 312, 132, 231 or 213 (all combinations except 123) meaning that there will be a specific change in the order of the notes between the first and the second *pitch pattern*. The task of the participant is to determine, whether the *visual number code*, that represents the manipulation of the order of the notes of the first *pitch pattern*, corresponds to the order of the notes of the second pattern. For instance if the tonal pattern is reversed, i.e. the notes are played in reversed order in the second pattern, and the *visual number code* provides the numbers 321, he/she should respond “correct” (left button), if the order of the notes of second *pitch pattern* does not correspond to the *visual number code*, they should respond “incorrect” (right button).

In the ***control condition*** participants listen to a *pitch pattern* consisting of 3 beeps of identical frequency/pitch, followed by a *visual number code* of 3 identical numbers, either 111, 222 or 333. They then receive an instruction on the computer screen whether they have to push the right or the left button of the response pad.

#### Bloodsampling

In addition, we collect one blood sample (10 ml) of each consenting participant (until now most people consented), close to each MRI measurement (t0, t1, t2, t3), for analyzing genotypes (only once) and methylation status (all times) of specific neuronal plasticity and learning related parameters (e.g. brain derived neurotrophic factor (BDNF), dopamine, sertonine, protein phosphatase 1, reelin and some others).

#### Procedures

A Flowchart of the study procedures with timelines is provided in Table 6.

**Table 6:** Study procedure with timelines

RECRUITEMENT & PRE-screening					
Months	Stage	What	Where	Action	Duration
MONTH 1–3	Preparatory phase	Recruitment of participants (n = ~ 70 in GE; n = ~ 100 in HA, on a total of ~ 500 applications) according to exclusion/exclusion criteria Detailed preparation of Behavioral Battery (psychometric testing) & (f)MRI protocols	Local journals/ elderly journals/ flyers/presentations HEdS-GE^2^ & IMMM^3^ BBL^5^ / HMS^6^	Announcements (press & flyers) Presentations^1^/1rst check by telephone Creation of E-prime^4^ programs; preparation of paper and pencil tests; Synchronization of protocols between MRI scanners in Geneva and Hannover	
Month 4		Piloting of (f)MRI protocols (*N* = 20) & Behavioral Battery (*N* = 10) on non-included elderly individuals	BBL / HEdS-GE / HMS / IMMM	Feasibility testing	
	Pre-screening Step 1	See Table 2	At home	Send/receive (mail/post)	60 min.
Month 5–6	Pre-screening Step 2 (N GE = 63, N HA = 92)	Check/completion of questionnaires of Step 1 Basic clinical audition test Psychometric tests i. Right-handedness ii. COGTEL	HEdS-GE/ IMMM	Come to HEdS-GE, IMMM 1x	20 min. 2 min. 5 min. 15 min.
After inclusion in the study					
Month 7–10		Recruitment and training of music teachers Preparing of music education materials			
	t0/baseline	i. Behavioral Battery (psychometric testing) ii. (f)MRI measurements iii. Blood sampling (by registered nurse)	HEdS-GE / IMMM BBL / HMS HEdS-GE / IMMM	Come to HEdS-GE, IMMM 1x Come to BBL, HMS 1x Come to HEdS-GE, HMS 1x	2.5 h^7^ 1.5 h 10 min.
Month 10–22	Interventions	12 months of 1 h weekly music courses & homework	HEdS-GE / IMMM At home	HEdS-GE / IMMM Practice daily at home	1 h/week 5 × 30 min./week
MONTH 16	t1/ after 6 months of intervention	i. Behavioral Battery (psychometric testing) ii. (f)MRI measurements iii. Blood sampling	HEdS-GE / IMMM BBL / HMS HEdS-GE / HMS	Come to HEdS-GE, IMMS 1x Come to BBL, HMS 1x	2.5 h 1.5 h 10 min.
Month 22	t2/directly after intervention completion	i. Behavioral Battery (psychometric testing) ii. COGTEL iii. (f)MRI measurements iv. Blood sampling v. Food Frequency Questionnaire vi. Cognitive reserve index1 (CRIq)^8^ [116]	HEdS-GE / IMMM HEdS-GE / IMMM BB / HMS HEdS-GE / HMS At home HEdS-GE / IMMM	Come to HEdS-GE, IMMM 1x (same session as Behavioral Battery) Come to BBL, HMS 1x Come to HEdS-GE, HMS 1x Send (mail/post) Come to HEdS-GE, IMMM 1x	2.5 h 15 min. 1.5 h 10 min. 30 min. 15 min.
Month 23–26	Cope with Delays, Analyses & Publications, Congress Attending				
Month 27	t3/6 months after intervention completion	i. Behavioral Battery (psychometric testing) ii. (f)MRI measurements iii. Blood sampling	HEdS-GE / IMMM BBL / HMS HEdS-GE / HMS	Come to HEdS-GE, IMMM 1x Come to BBL, HMS 1x Come to HEdS-GE, HMS1x	2.5 h 1.5 h 10 min.
Month 28–36	Cope with Delays, Analyses & Publications, Congress Attending				

^1^Presentations at Cité Séniors Genève (http://www.ville-geneve.ch/themes/social/seniors/cite-seniors/); at the HEdS-GE^2^ and in Hannover at the HMTMH (Hannover University of Music Drama and Media)

^2^ HEdS-GE Haute école de Genève, 47 av. de Champel 47, 1206 Genève

^3^ Institute of Music Physiology and Musicians’ Medecine, Hannover, part of the HMTMH

^4^ Presentation soft-ware (https://pstnet.com/products/e-prime/)

^5^ Brain and Behaviour Laboratory (BBL, http://bbl.unige.ch/) , University Medical Centre of the University of Geneva

^6^ Hannover Medical School

^7^ During the Behavioral Battery several short breaks are included

^8^ This test has been added posteriorly

#### Additional questionnaires

##### Cognitive reserve index questionnaire (CRIq)

The CRIq [116] has been added posthoc to the testing. It measures cognitive reserve built up over the lifespan. It will be added to the Behavioral Battery at t2 (12 months, see Table 6). Cognitive reserve is the resilience to neuropathological damage in older adults, as a consequence of life-long learning and experiences.

##### Food frequency questionnaire FFQ

The FFQ [91] is used during the pre-screening. It consists of a semi-quantitative questionnaire existing in French and German, on 80 food items divided in 12 food groups. We want to investigate the potential links between quality and quantity of food intake, learning, cognitive functioning, general health and brain function and structure.

#### Progress measures

In order to evaluate the progress in both groups following training, we gather the following measures:

##### Piano group

- Evaluation at 3, 6 and 12 months after intervention onset by the teachers, and at 3 and 12 months after intervention onset by professional musicians that do not follow the courses otherwise. The participants are evaluated on 7 different 5-point Likert scales, concerning 1) technique, 2) rhythm, 3) expressivity, 4) text compliance / music reading, 5) ensemble playing, 6) motivation / homework and 7) general progress.
- A simplified version of Beethoven’s Ode to joy, to be played with both hands on the piano, is recorded (MIDI) after a 2 week preparation at 3 months, at 12 months (end of training) and at 18 months (6 months after course completion).
- Slow up-and-down playing of a scale of 5 notes at a pace of 76 beats per minutes indicated by a metronome, with 1 note per beat, c-d-e-f-g (so no position change) at the central octave of the piano is recorded (MIDI) at t0, t1, t2 and t3.
- Fast up-and-down playing of a scale of 5 notes at 76 beats per minute, with 2 notes per beat, c-d-e-f-g (so no position change) at the central octave of the piano is recorded (MIDI) at t0, t1, t2 and t3.
- The “Genie test”, where participants press the same note (central C) 5 times in crescendo, i.e. increasing the loudness note after note, and 4 times in decrescendo, decreasing the loudness note after note at t0, t1, t2 and t3 (no rhythm to follow)

##### Musical culture / control group

- Evaluation at 3, 6 and 12 months after intervention onset by the teachers, and at 3 and 12 months after intervention onset by professional musicians that do not follow the courses otherwise. The participants are evaluated on 6 different 5-point Likert scales, concerning 1) group ambiance, 2) individual participation, 3) interaction (individual), 4) joy (individual), 5) motivation / homework (individual), 6) general progress (individual).
- A multiple choice questionnaire involving musical knowledge and listening is passed at t0, t1, t2 and t3
- Slow up-and-down playing of a scale of 5 notes at a pace of 76 beats per minutes indicated by a metronome, with 1 note per beat, c-d-e-f-g (so no position change) at the central octave of the piano is recorded (MIDI) at t0, t1, t2 and t3.

Both groups also complete a training amount questionnaire at 3, 6, 9 and 12 months (see Table 3 “Homework questionnaire”) of intervention, indicating how much time per day and per week each participant spent on homework on average over the last 3 months. These progress and training amount measures will be linked in the final analyses to other brain and behavioral measures.

### Data monitoring and quality assurance

At each site data acquisition and quality is constantly monitored by experimenters for each participant (Damien Marie and Laura Abdili in Geneva, Florian Worschech, Kristin Jünemann and Christopher Sinke in Hannover). Progress meetings (by Skype) are organized regularly between both sites. A second quality check is performed on the server where all data are backed up (see **Declarations,** Availability of data and materials, p. 16) and analyzed by Damien Marie (see Declarations p. 16). A third checkup is performed by an independant Data Manager (scientific collaborator at PhD level) both at the the HEdS-GE and the HMTMH, after each data point.

We will report reasons for withdrawal of individuals for each randomization group. Exact information on missing data at each time point will be reported in all publications. Missing data will be processed in the analyses with cutting edge means (for instance using the regularized iterative Principal Component Analysis (PCA) algorithm developed by Josse, Husson and Pagès (2009) [117], implemented in the R package missMDA.

### Statistical analyses

First, we will study cross-sectionally all relationships between behavior and brain data collected at baseline (data from t0) for all participants (1) based on a priori hypotheses using general linear models, correlations and regression analyses; (2) Using data-driven multivariate approaches that allow to reveal more intricate relationships between the data.

Brain data will mostly be analyzed using in-house script routines calling the last versions of the standard neuroimaging software pipelines (SPM12, FSL6, FreeSurfer, MRtrix etc.), all running on the same operating system on our server (Linux xfce4.12) to avoid any potential operating system bias [118].

Second, we will examine and compare the development in both experimental groups over time (t0 vs. t1 vs. t2 vs. t3) for the comprehensive set of behavioral and brain data using univariate and multivariate methods. Individuals’ intensity of training, progress, demographic characteristics and post-hoc appreciation of the courses will be taken into account (implemented as co-variates).

Analyzing separately behavioral data and several kinds of brain imagery data cross-sectionally or over time provides valuable information on these specific data. However, blending diverse behavioral and different kinds of brain data within data-driven multivariate analyses may unravel hidden “covert” relationships [119, 120]. For these advanced analyses we will be assisted by Prof. D. Van De Ville, computer scientist; expert in advanced (f)MRI data analyses).

### Detailed information on multivariate analyses

In close collaboration with Prof. Van De Ville and his team of data scientists, we will perform final analyses on our data using multivariate data-driven techniques such as multimodal Independent Component Analysis (ICA) [119, 120] and Partial Least of Squares (PLS) [121, 122], which have both been used successfully on multimodal brain imagery data. These techniques allow to (1) significantly reduce the dimensionality of the data (as compared to a voxel-by-voxel analysis), reducing multiple comparison correction; (2) improve the interpretability by identifying brain regions or networks that can be more easily related to known processes. Two types of methods will be used:

1) Joint, fusion, and linked Independent Component Analysis (ICA) [123, 124] represent several blends of multimodal extensions of ICA that allow combining different types of data such as functional and structural neuroimaging data. Depending on the type of ICA, weaker or stronger relationships between the modalities are assumed. In addition, we will also decompose the fMRI data into functional brain networks using innovation-driven co-activation patterns (iCAPs), a recent approach of dynamic functional connectivity [125] that can quantify temporal interactions between different networks. The results of these analyses should reveal interactions between different types of information and consequently allow us to investigate differences between our groups [119, 123].

2) Partial Least of Squares (PLS) is another powerful technique that can identify components of multivariate relationships between imaging and behavioral data [122, 126].

Implementations of these approaches (1 & 2) are publicly available in addition to a number of in-house extensions that allow using them in the most flexible way, including machine-learning approaches [127, 128].

#### Power analyses

No standardized computational tools exist for the power analysis of longitudinal neuroimaging studies [129]. A true longitudinal design provides increased statistical power reducing the confounding effects of between-subject variability [130]. Foreseeing an important attrition of 25% in our elderly population, we recruited ~ 30 participants per experimental and per control group in Geneva, and ~ 45 individuals per group in Hannover, so a total of 150 participants. Anticipating around 25% of attrition, final analysis will thus be performed in principle on more than 112 participants, ~ 56 in the experimental and ~ 56 in the control groups.

In order to acquire sufficient statistical power to reliably detect a 2% difference in Gray Matter volume [36] between 2 groups at the whole brain level, a longitudinal study design requires on average 38 subjects, equally divided between the 2 groups [131]. Colcombe and colleagues [81] found reliable frontal GM and WM (White Matter) differences after only 6 months of aerobic training in elderly with much smaller sample sizes (n total = 59, divided evenly over an experimental and a control group) than in this study. To detect reductions in mean tract FA (Fractional Anisotropy, reflects fiber density, measure resulting from Diffusion Tensor Imaging (DTI)) of 2–20%, sample sizes between 7 and 28 subjects are recommended for cross-sectional analyses for an effect size of 5%, depending on the white matter tracts studied [132], but longitudinal studies are more powerful.

Longitudinal intervention studies with 150 participants are rare. All the cited studies involved smaller sample size compared to the current study. Therefore, we consider to have opted for a fair compromise between feasibility of the study and number of participants.

### Outcomes of the study

#### Primary outcomes

We expect positive transfer effects from intensive piano training (intervention group) - as compared to listening and learning about music without practice (control group) - over one full year on age-related cognitive decline: for working memory, executive functions, speed of information processing, auditory selective attention (hearing in noise) and abstract thinking. Associated functional and structural brain plasticity may show in gray and white matter in temporal (medial and lateral), prefrontal and parietal areas. We specifically anticipate - in connection with these cognitive changes - less decline or increase of gray matter volume and activation in the hippocampus, and improved functional networking in frontal areas during working memory tasks.

##### Secondary outcomes

 Benefits for fine perceptual-motor skills may also manifest in the intervention group as compared to the control group associated with functional and structural brain plasticity in sensorimotor areas.

##### Tertiary outcomes

Subjective quality of life, verbal memory and hearing in noise may improve in both groups, the latter two associated with functional and structural brain plasticity in auditory and frontal areas.

##### Quaternary outcomes

Specific results of epigenetic changes may be induced by piano learning as a specific form of exercise and show a relationship with functional and structural imaging parameters.

##### Control outcome

Higher scores for the musical tests (GOLD MSI for rhythm and melody, Pietri task for harmony (see Table 4)) in the piano group as compared to control group.

### Broader impact

If this applied research study is successful, it may serve as a springboard for the development and implementation of targeted music practice interventions for different types of healthy elderly and patients in the context of population aging and lifespan development, prolonging autonomy, serving public health and thus reducing health costs.

### Study specific risks/ inconveniences

#### Risks of (f)MRI scanning

(f)MRI scans are non-invasive and yield few potential risks because they do not involve any radiation, only a strong magnetic field. A strictly protocolled MRI safety procedure will be applied in both labs (Hannover: Department of Neuroradiology of the Hannover Medical School, Geneva: Brain and Behaviour Laboratory of the University of Geneva), verifying for any metal objects outside and inside the body, as well as for special conditions (pacemaker, etc.).
People not comfortable with (f)MRI scanning will be excluded from the experiment.
Full information will be provided on the (f)MRI technique beforehand, group visits of the MRI room will allow participants to verify whether they can cope with the MRI environment.

### Coverage of damages: insurance

Geneva:

Insurance « Responsabilité civile » de la Haute école de santé de Genève.

AXA Winterthur Police no. 4.626.347

Sum of insurance CHF 10′000’000.- Fixed sum per event, including bodily injury, property damage and insured expenses.

Hannover:

Insurance: Alte Leipziger, Police no 30–660–104-204 FD. Sum of Insurance Euro 3′000’000 Fixed sum per event, including bodily injury, property damage and insured expenses.

## Discussion

This multi-site study required harmonization between the Geneva and Hannover site concerning all musical interventions, tests, measurements and questionnaires.

We managed (see Table 4) to create an identical test battery at both sites, by choosing tests existing in French and German, but also assured by translations performed by several bilingual members of the team.

The musical education programs were also composed in very similar ways, assured by continuous exchange between the team of musical education experts.

Concerning MRI protocols, the challenge was to end up with corresponding MRI sequence parameters at both sites, allowing to pool the data with a mimimum of post-hoc adaptations. As 3 T Siemens scanners are present at both sites, blending the data is possible [115]. We managed to end up with identical sequence parameters (see Table 5). Even so, in all analyses, we will add a “scanner” covariate in order to disentangle potential scanner-effect from effects-of-interest.

Although some slight timing differences exists between the Geneva and Hannover sites, the protocols are identical and follow the same chronological path (see Table 6).

In this study, it is impossible for the experimenters to remain ignorant of group membership.

## Data Availability

As data collection is ongoing, measured GE and HA data are temporarily stored at a 18 To secured server hosted in a secured datacenter at the HES-SO Geneva (HEPIA: Haute Ecole du Paysage, d’Ingénieurie et d’Architecture), accessible to only a part of GE and HA team members via a double authentication VPN (first authentication through user password triggers the receipt of a second password by text message on the personal phone of the user) and encrypted ssh connection. The server back-up system has two levels. The first level concerns the hardware storage that is set up in RAID1 (Redundant Array of Independent Disks), each data file is duplicated on two separate hard-drives to prevent losing data because of a hard-drive failure. The second level consists of an encrypted copy of all data in another third-party datacenter following research ethics guidelines (automatic process performed every 3 h) The server will also be used for all analyses (Intel Xeon Gold 5115 2.4 GHz processor, 2 sockets * 10 cores * 2 Threads = 40 threads, 4*32Go RDIMM 2666MT/s Dual Rank126 Go of RAM) As soon as the data collection will be completed, all data and all other documents will also be stored at YARETA, a FAIR digital solution for long-term preservation of research data for all Geneva Universities (https://yareta.unige.ch). The HEdS-GE already possesses an organizational unit on the YARETA platform. The datasets generated and/or analyzed during the current study are not publicly available at present due to the fact that 1) the data are only partially collected, and 2) will be published by our research team first, but they will be available in the future from the corresponding author on reasonable request. Measured data are anonymized, the code system for the anonymization of the participants is only preserved at the respective GE and HA Institutes, secured by unique passwords, on 1 computer and 1 hard drive at each site, only accessible to Eckart Altenmüller, Clara James, Damien Marie & Christopher Sinke (Postdoctoral fellows GE & HA), Florian Worschech (PhD student HA) and one other PhD student (HA).

## Abbreviations

ASL: Arterial Spin Labeling
BMI: Body Mass Index
BBL: Brain and Behaviour Laboratory
(f)MRI: (functional) Magnetic Resonance Imaging
CRIq: Cognitive Reserve Index questionnaire
DFG: Deutsche Forschungsgemeinschaft
DTI: Diffusion Tension Imaging
EPI: Echo-Planar Imaging
FA: Fractional Anisotropy
FFQ: Food Frequency Questionnaire
FOV: Field Of View
GE: Geneva (Switzerland)
GEMMI Lab: Geneva Musical Minds Laboratory
COGTEL: Cognitive Telephone Screening Instrument
GM: Grey matter
HA: Hannover (Germany)
HES-SO: Haute Ecole Spécialisée de Suisse occidentale
HEdS-GE: Haute école de santé de Genève
HEM-GE: Haute Ecole de Musique de Genève
HEPIA: Haute Ecole du Paysage, d’Ingénieurie et d’Architecture
HMTMH: Hochschule für Musik, Theater und Medien Hannover
ICA: Independent Component Analysis
IMMM: Institut für Musikphysiologie und Musikermedizin
iCAPs: Innovation-Driven Co-Activation PatternS
ISBN: International Standard Book Number
MP2RAGE: Magnetization-Prepared 2 Rapid Acquisition Gradient Echoes
PLS: Partial Least of Squares
RAID1: Redundant Array of Independent Disks
SNSF: Swiss National Science Foundation
T: Tesla
t0: Baseline measurement
t1: 6-months measurement
t2: 12-months measurement
t3: 6-months post-intervention measurement
TI1: First inversion time
TI2: Second inversion Time 2
TE: Time of Echo
TED: Technology, Entertainment and Design
TR: Time of Repetition
VBM: Voxel Based Morphometry
WHOQOL-BREF: World Health Organization Quality of Life-BREF questionnaire
WM: White matter

## Footnotes

1 Cognitive Telephone Screening Instrument. This cognitive screening instrument is a test of global cognition that can be applied by telephone or face-to-face (we applied face-to-face; see Table 2).

2 The keyboards were generously offered by Yamaha Germany and Yamaha Switzerland. Their generous gift included instruments for the classrooms and for each piano group participant to take home.

3 Culture musicale (GE; French nomenclature) / Musik erleben und verstehen (HA; German nomenclature)

4 Test-retest effects are learning effects between time points of data collection
